# Intrinsic nonlinear geometric phase in SHG from zincblende crystal symmetry media

**DOI:** 10.1515/nanoph-2024-0162

**Published:** 2024-06-26

**Authors:** Luca Carletti, Davide Rocco, Maria Antonietta Vincenti, Domenico de Ceglia, Costantino De Angelis

**Affiliations:** Department of Information Engineering, Univeristy of Brescia, Via Branze 38, 25123, Brescia, Italy; National Institute of Optics – National Research Council (INO-CNR), Via Branze 45, 25123, Brescia, Italy

**Keywords:** second harmonic generation, nonlinear nanophotonics, nonlinear metasurfaces, nonlinear geometric phase, wavefront shaping metasurfaces

## Abstract

We demonstrate that AlGaAs thin films and metasurfaces generate a distinct intrinsic nonlinear geometric phase in their second harmonic signals, differing significantly from previous studies on nonlinear dielectric, plasmonic, or hybrid metasurfaces. Unlike conventional observations, our study reveals that the second harmonic phase remains unaffected by the linear optical response at both pump and harmonic wavelengths, introducing a novel realm of achievable phase functions yet to be explored. Furthermore, we explore the interplay between this intrinsic nonlinear geometric phase and the geometric phase induced by rotations of nanoresonators within metasurface arrangements. Our findings extend the capabilities of nonlinear wavefront shaping metasurfaces, exploiting phase manipulation to uncover unique phenomena exclusive to the nonlinear regime.

## 1 Introduction

Optical metasurfaces, comprised of subwavelength arrangements of optically resonant nanostructures, present an advanced approach to design novel optical components for modern applications [1], [2], [3], [4], [5]. By employing meticulously engineered nanostructures, metasurfaces enable precise control over the phase, amplitude, and polarization of light in a highly compact and unique manner that surpasses the capabilities offered by refractive lenses. The precise modulation of phase at a subwavelength scale is essential to realize numerous optical functions, including imaging [6], [7], focusing [8], [9], digital holography [10], and all-optical multiplexing [11], [12]. Within this framework, Pancharatnam–Berry phase metasurfaces (PB-metasurfaces) streamline the engineering of wavefronts by adjusting the linear phase of transmitted or reflected light through localized nanostructure rotation [1], [11], [12], [13]

In the last decade, the use of engineered nanostructures and metasurfaces has also emerged for the manipulation of nonlinear optical processes [14], [15]. This is due to their great potential to reduce the size of devices while adding exotic functionalities. Relevant examples include nonlinear plasmonic metasurfaces and photon-spin dependent nonlinear geometric-phase structures [16], [17], [18]. Additionally, hybrid metasurfaces, integrating plasmonic structures with semiconductor quantum wells, have been utilized to effectively control nonlinear wavefront [19]. Recent research has also investigated the selective behavior of dielectric nanostructures in nonlinear harmonic generation processes [20], [21], [22]. All-dielectric metasurfaces present in fact a promising alternative to their plasmonic-based counterparts, capitalizing on Mie resonances to mitigate dissipation losses and enhancing durability and efficiency, thus making them attractive for optical wavefront manipulation. However, in all these demonstrations, the geometrical phase is typically imparted through the linear optical response of the pump and then passed onto the generated harmonics, leading to increased design complexity and constraining the range of potential applications.

In this study, we investigate how the rotation of the crystal axes of a thin film of AlGaAs affects the phase and amplitude of second-harmonic generation (SHG) induced by circularly polarized light of opposite chirality. While the material appears isotropic from a linear perspective, the observed geometric phase stems from the anisotropy of the second-order nonlinear tensor, impacting solely the second harmonic (SH) signal. Thus, this phenomenon represents a purely nonlinear geometric phase effect. By altering the crystal cut and symmetries, various SHG phase functions can be attained, enhancing the current capabilities for phase manipulation. Additionally, we explore the impact of the nonlinear geometric phase in a metasurface scenario: by combining crystal axes and resonators’ rotations we can observe a novel phase function that has not been previously observed. Our findings broaden the scope of dielectric metasurfaces for nonlinear wavefront manipulation by introducing new phenomena specific to the nonlinear regime.

## 2 Results and discussion

**Figure 1:**
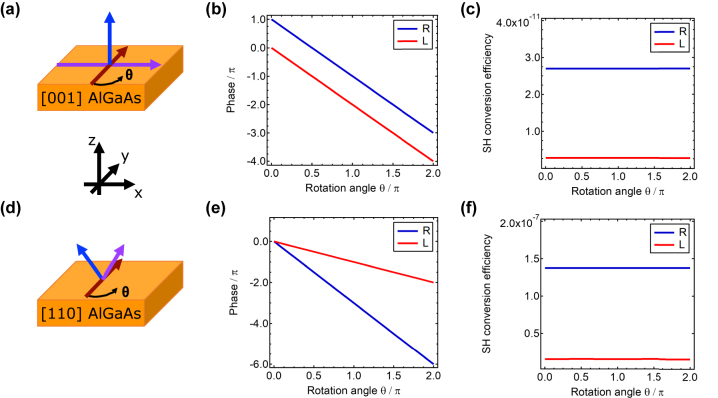
SHG from AlGaAs thin-film. (a) Schematic representation of the [001] AlGaAs thin film geometry. The crystal axes ([100], [010], and [001] are represented as purple, red and blue arrows, respectively) and in-plane rotation angle *θ* are depicted. (b) Phase and (c) conversion efficiency of L and R polarized SH emitted from the film in the forward direction with respect to the L polarized pump beam as a function of the in-plane crystal rotation *θ*. (d) Schematic representation of the [110] AlGaAs thin film geometry. (e) Phase and (f) conversion efficiency of L and R polarized SH emitted from the film in the forward direction with respect to the L polarized pump beam as a function of the in-plane crystal rotation *θ*.

The general concept of the intrinsic nonlinear geometric phase is schematically represented in Figure 1(a). A thin layer of [001] AlGaAs is illuminated by a plane wave at a wavelength of 1,550 nm with a circularly polarized electric field. The crystal is rotated by an angle *θ* and the amplitude and phase of the emitted second harmonic (SH) is observed. To investigate the relation between the rotation angle *θ* and the phase of the emitted SH, we recast the second-order nonlinear tensor of AlGaAs in circular polarization (CP) basis $\chi_{\alpha \beta \gamma }^{\left(2\right)}$ , where *α*, *β*, and *γ* can be R, L, or Z, corresponding to right-handed (R), left handed (L) circularly polarized and linearly polarized along the *z*-axis electric fields, respectively. Once the *χ* ^(2)^ tensor is expressed in this form, it might be straightforward to predict the geometric phase acquired by the SH. For a [001] AlGaAs thin film, with crystal axes aligned with the reference Cartesian coordinate system, with *θ* = 0 rad the *χ* ^(2)^ tensor has the following components (see Section S1 in the Supporting Information)

(1) $$\chi_{\text{RLZ}}^{\left(2\right)}=-\chi_{\text{LRZ}}^{\left(2\right)}=-\chi_{\text{ZRR}}^{\left(2\right)}=\chi_{\text{ZLL}}^{\left(2\right)}$$

Considering the SH emitted in transmission (i.e., on the opposite side of the thin film with respect to the incident pump), the nonlinear polarization due to SHG in the CP basis, after the rotation of *θ* of the crystal is (see Section S1 in the Supporting Information)

(2) $$P_{R}\left(2\omega \right)=\varepsilon_{0}\chi_{\text{RLZ}}^{\left(2\right)}2E_{L}^{\omega }E_{z}^{\omega }{\text{e}}^{-\text{i}2\theta }$$

(3) $$P_{L}\left(2\omega \right)=\varepsilon_{0}\chi_{\text{LRZ}}^{\left(2\right)}2E_{R}^{\omega }E_{z}^{\omega }{\text{e}}^{\text{i}2\theta }$$

(4) $$P_{Z}\left(2\omega \right)=\varepsilon_{0}\left[\chi_{\text{ZRR}}^{\left(2\right)}{\left(E_{R}^{\omega }\right)}^{2}{\text{e}}^{\text{i}2\theta }+\chi_{\text{ZLL}}^{\left(2\right)}{\left(E_{L}^{\omega }\right)}^{2}{\text{e}}^{-\text{i}2\theta }\right]$$

We note that the phase factor induced by the crystal rotation is e^±i2*θ*^, which is different from the conventional nonlinear geometric phase observed in other structures, in which the polarization phase follows the typical e^*σ*i(*N*±1)*θ*^ factors, where *N* is the harmonic number and *σ* = ±1 for R or L pump [17], [19]. In fact, the phenomenon described by Eqs. (2)– (4) is induced only by the anisotropy of the second-order tensor that mixes different electric field components.

To illustrate the aforementioned effect, we used frequency-domain simulations implemented with the finite-element-method in Comsol Multiphysics (see Section S2 in the Supporting Information). The thickness of the film is arbitrarily fixed to 400 nm. The choice of this geometrical parameter is irrelevant if its value is small enough compared to the pump and SH wavelengths so that phase-matching can be neglected. The film is considered to be infinitely extended in the *xy*-plane. Due to symmetry selection rules [23], [24], the pump has a 1° angle of incidence, with respect to the film normal direction. This breaks the in-plane symmetry and allows a non-zero SH far-field radiation. The conversion efficiency (defined as the ratio of the intensities of the SH and pump [25]) and phase of the transmitted SHG as a function of the crystal rotation angle *θ* are shown in Figure 1(b) and (c), respectively. As we can observe, the phase of the SHG follows a −2*θ* dependency. The electric field in the thin-film is predominantly $E_{L}^{\omega }$ with a very weak $E_{Z}^{\omega }$ component induced by the off-normal incidence. Thus, the leading nonlinear polarization term from Eq. (4) is $P_{Z}\left(2\omega \right)=\varepsilon_{0}\chi_{\text{ZLL}}^{\left(2\right)}{\left(E_{L}^{\omega }\right)}^{2}{\text{e}}^{-\text{i}2\theta }$ . The non-zero angle of incidence introduces a small $P_{R}\left(2\omega \right)$ that allows the SH radiation to be observed in the far-field. On the other hand, the SH efficiency is constant at any angle *θ* as the geometric phase factor appears only in the nonlinear process, while no effect is observable at the pump frequency since the permittivity of the material is isotropic (i.e., a diagonal matrix with equal elements).

These results allow us to anticipate that if the crystal axes are rotated or other crystal symmetries are considered, different nonlinear geometric phase functions will be observed, therefore significantly enriching the panorama of nonlinear phase manipulations that can be realized. For example, the conversion efficiency and phase of the SH transmitted by a [110] oriented AlGaAs thin film (see Figure 1(d)) from a plane wave pump with L polarization at normal incidence is shown in Figure 1(e) and (f). Here we can observe that the co-polarized SH brings a *θ* geometric phase, while the cross-polarized SH varies as 3*θ*. Indeed, if we express the SH nonlinear polarization in CP basis for an L polarized pump at normal incidence, we have that the nonlinear SH polarization as a function of the rotation angle *θ* is (see Section S1 in the Supporting Information)

(5) $$P_{R}\left(2\omega \right)=\frac{\varepsilon_{0}\chi^{\left(2\right)}}{2\sqrt{2}}\left[3{\text{e}}^{-\text{i}3\theta }{\left(E_{L}^{\omega }\right)}^{2}\right]$$

(6) $$P_{L}\left(2\omega \right)=\frac{\varepsilon_{0}\chi^{\left(2\right)}}{2\sqrt{2}}\left[{\text{e}}^{-\text{i}\theta }{\left(E_{L}^{\omega }\right)}^{2}\right]$$

(7) $$P_{Z}\left(2\omega \right)=0$$

from which we can recognize a phase factor of e^−i3*θ*^ for R polarized SH in Eq. (5) and a factor e^−i*θ*^ for L polarized SH in Eq. (6). This is in agreement with our results in Figure 1(e) and it shows the same behaviour between SH phase and rotation angle of previous demonstrations in hybrid plasmonic multi quantum wells PB-metasurfaces [19]. Remarkably, in our study this effect is obtained without any nanostructure and from a linearly isotropic material.

**Figure 2:**
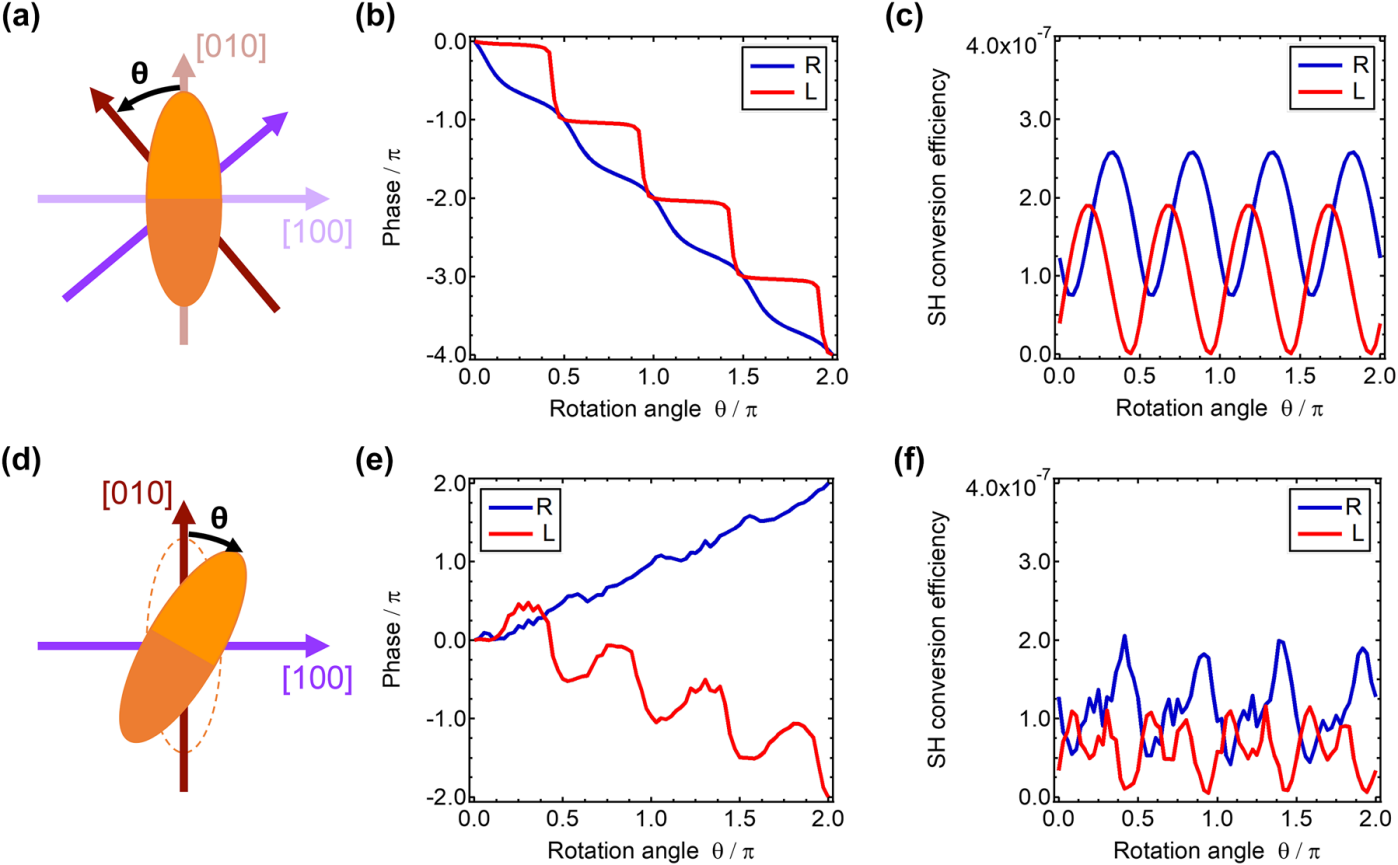
Linear and nonlinear geometric phase. (a) Schematic representation of the top-view of a metasurface unit-cell constituted by a nano-chair with elliptical cross-section (see Section S3 in the Supporting Information). The in-plane [100] and [010] crystal axes are represented by the purple and red arrows, respectively. The crystal axes after a rotation of *θ* are represented by the darker axes. (b) Phase and (c) SH conversion efficiency in transmission with respect to the L pump beam as a function of *θ*. (d) Schematic top-view of a metasurface unit-cell and rotation of the nano-chair by *θ*. (e) Phase and (f) SH conversion efficiency in transmission with respect to the L pump beam as a function of the in-plane rotation *θ*.

The intrinsic nonlinear geometric phase also plays a key role when the nonlinear material is structured into nanoresonators or arranged into metasurfaces. In this case, the linear geometric phase due to nanoresonator rotations adds up to the intrinsic nonlinear geometric phase. To illustrate this interaction let us start from the study of a metasurface with a quadratic unit-cell and where the orientation of the crystal axes with respect to the lattice is changed. This situation is represented in Figure 2(a). The nanoresonator is a nano-chair and is constituted by [001] AlGaAs [26], [27] (see Section S3 in the Supporting Information). This geometry guarantees efficient SH emission in the direction with respect to the metasurface plane through symmetry breaking. The nonlinear polarization in the nanoresonator can be expressed in the CP basis using Eqs. (2)– (4). Figure 2(b) shows the phase of the emitted SH as a function of *θ* that follows the same relation observed in the [001] AlGaAs thin film (Figure 1(b)). This is due to the dominant $P_{Z}\left(2\omega \right)$ nonlinear polarization component in the nano-chair (as happens in the thin-film scenario). On the other hand, the phase of the transmitted pump beam is insensitive to the rotation of the crystal because the dielectric permittivity tensor of AlGaAs is isotropic (see Section S4 in the Supporting Information). Additionally, from Figure 2(c), we observe that the SH conversion efficiency varies as a function of *θ*. This is due to the change of coupling efficiency between the induced nonlinear currents in the nano-chair and the optical modes of the metasurface. If the metasurface was polarization-independent, this variation would not occur. The interaction between the different geometric phase processes could be exploited to realize a nonlinear wavefront shaping metasurface organized in pixels, each constituted by the same array but with an orientation guided by the target phase response. In such a device, the wave-shaping function would be transparent to the pump frequency and only effective for the SH light. This feature is not achievable with the current nonlinear PB-metasurfaces paradigm.

As last example, we consider a rotation of the nano-chair orientation while the crystal axes are fixed as represented in Figure 2(d). This scenario corresponds to the typical nonlinear PB-metasurfaces [17], [18], [19] and it can be realized with standard nanofabrication techniques since it does not involve rotation of the film crystallographic axes. Figure 2(e) shows the phase of the transmitted L and R SH as a function of the rotation angle *θ*. We observe that the phase of L polarized SH is proportional to *θ* while the phase of R polarized SH is proportional to −*θ*. These relationships can be predicted by considering the combined effect of the relative rotations of the nano-chair and the crystal axes. Let us note that, with respect to the original system of reference, they rotate in opposite directions. Thus, in addition to the intrinsic nonlinear geometric phase, a linear geometric phase generated by the rotation of the polarization-sensitive unit-cell must be considered, similar to the effect at the pump frequency (see Section S4 in the Supporting Information). The SH with the same polarization of the pump will acquire a e^*σ*i*θ*^ factor, while the cross-polarized SH will acquire a e^*σ*i3*θ*^ phase factor, with *σ* = ±1 for R or L polarized pump, respectively [17], [19]. Thus, in our metasurface, the R polarized SH acquires a factor e^−i2*θ*^ due to the crystal rotation and e^−i3(−*θ*)^ from the lattice rotation, with a net total phase factor of e^i*θ*^. Likewise, the L polarized SH acquires a factor e^−i2*θ*^ due to the crystal rotation and e^−i(−*θ*)^ from the lattice rotation, for a total phase factor of e^i*θ*^. This is unique to the bulk SHG process in the nonlinear crystal, and it is not reproducible using surface nonlinearities as in metals. Furthermore, different functions might be obtained by varying the crystal or lattice symmetry. Finally, we observe that the conversion efficiency shown in Figure 2(f) also varies as a function of *θ*. This is again an effect of the polarization-sensitive response of the metasurface. Note that, differently from typical PB-metasurfaces, a polarization-sensitive metasurface is not necessary for the intrinsic nonlinear geometric phase to be observed.

## 3 Conclusions

We numerically demonstrate a novel nonlinear geometric phase principle that allows to enrich the phase control of the generated SH with respect to the already reported strategies. Differently from commonly employed schemes, our approach is not dictated by the response of the structure at the pump wavelength, thus transferring the geometric phase only onto the nonlinearly generated light. Finally, by playing with different crystal axes rotations or by considering materials with different nonlinear susceptibility tensors, we can easily envision how this method allows to implement a wide set of new phase functions that have never been reported before in the literature. Our results unveil a new mechanism for advanced manipulation of the waveform generated through nonlinear optical processes in crystals and may guide the development of novel nonlinear dielectric metasurfaces for new applications.

## Supporting Information

Nonlinear permittivity tensor of zinc-blende crystals in circular polarization basis; Numerical simulations; Nano-chair metasurface geometry.

## Supplementary Material

Supplementary Material Details
