# Supplementary material for: Intrinsic nonlinear geometric phase in SHG from zincblende crystal symmetry media
Source: Nanophotonics. 2024 Jun 26;13(18):3321–6. doi: 10.1515/nanoph-2024-0162 (PMC11501106; doi:10.1515/nanoph-2024-0162)
Supplement: Supplementary file 1 — Supplementary Material Details [file j_nanoph-2024-0162_suppl_001.pdf]

Intrinsic nonlinear geometric phase in SHG from zincblende crystal symmetry media

L. Carletti,*,†,‡ D. Rocco,†,‡ M. A. Vincenti,†,‡ D. de Ceglia,†,‡ and C. De Angelis†,‡

†*Department of Information Engineering, Univeristy of Brescia, Via Branze 38, 25123, Brescia, Italy*

‡*National Institute of Optics - National Research Council (INO-CNR), Via Branze 45, 25123, Brescia, Italy*

E-mail: luca.carletti@unibs.it

S1 Nonlinear permittivity tensor of zinc-blende crystals in circular polarization basis

Let us assume a plane wave at angular frequency ω propagating towards the $\pm z$-axis direction. We perform a coordinate transformation between the linear polarization (LP) and the circular polarization (CP) basis formed by:

$$\hat{e}_L^{\pm} = \frac{1}{\sqrt{2}} \left(\hat{e}_x \pm i \hat{e}_y \right) \tag{S1}$$

$$\hat{e}_R \pm = \frac{1}{\sqrt{2}} \left(\hat{e}_x \mp i \hat{e}_y \right) \tag{S2}$$

$$\hat{e}_Z = \hat{e}_z \tag{S3}$$

The transformation matrices between the two bases are:

$$\bar{\bar{\Lambda}}^{\pm}_{\alpha,k} = \frac{1}{\sqrt{2}} \begin{bmatrix} 1 & \pm i & 0 \\ 1 & \mp i & 0 \\ 0 & 0 & \sqrt{2} \end{bmatrix}, \qquad \bar{\bar{\Lambda}}^{\pm}_{k,\alpha} = \frac{1}{\sqrt{2}} \begin{bmatrix} 1 & 1 & 0 \\ \mp i & \pm i & 0 \\ 0 & 0 & \sqrt{2} \end{bmatrix} \tag{S4}$$

Any electric field polarization expressed in the Cartesian coordinate system can thus be represented in the CP basis as:

$$E_k = \sum_{\alpha} \lambda_{k,\alpha} E_\alpha \tag{S5}$$

where $\lambda_{k,\alpha}$ are the elements the transformation matrix in Eq. S4. The nonlinear polarization in the CP basis for radiated waves propagating in the positive z-axis direction is:

$$P^{2\omega}_{\alpha} = \sum_{k} \lambda^{+}_{\alpha,k} P^{2\omega}_{k} = \varepsilon_0 \sum_{k,j,s} \lambda^{+}_{\alpha,k} \chi^{(2)}_{kjs} E^{\omega}_{j} E^{\omega}_{s} \tag{S6}$$

After substituting S5 into S6, one obtains:

$$P^{2\omega}_{\alpha} = \varepsilon_0 \sum_{\beta,\gamma} \sum_{k,j,s} \lambda^{+}_{\alpha,k} \chi^{(2)}_{kjs} \lambda^{+}_{j,\beta} \lambda^{+}_{s,\gamma} E^{\omega}_{\beta} E^{\omega}_{\gamma} \tag{S7}$$

from which we can express the nonlinear tensor in the CP basis as:

$$\chi^{(2)}_{\alpha\beta\gamma} = \sum_{k,j,s} \lambda^{+}_{\alpha,k} \chi^{(2)}_{kjs} \lambda^{+}_{j,\beta} \lambda^{+}_{s,\gamma} \tag{S8}$$

Now, let us consider AlGaAs. The nonlinear susceptibility tensor has only non-zero components of the type $\chi^{(2)}_{kjs}$ with $k \neq j \neq s$. From Eq. S8 we thus obtain that the only non-zero

components are:

$$\chi^{(2)}_{RLZ} = \lambda^{+}_{R,x}\chi^{(2)}_{xyz}\lambda^{+}_{y,L}\lambda^{+}_{z,Z} + \lambda^{+}_{R,y}\chi^{(2)}_{yxz}\lambda^{+}_{x,L}\lambda^{+}_{z,Z} = i\chi^{(2)} \tag{S9}$$

$$\chi^{(2)}_{LRZ} = \lambda^{+}_{L,x}\chi^{(2)}_{xyz}\lambda^{+}_{y,R}\lambda^{+}_{z,Z} + \lambda^{+}_{L,y}\chi^{(2)}_{yxz}\lambda^{+}_{x,R}\lambda^{+}_{z,Z} = -i\chi^{(2)} \tag{S10}$$

$$\chi^{(2)}_{ZRR} = \lambda^{+}_{Z,z}\chi^{(2)}_{zxy}\lambda^{+}_{x,R}\lambda^{+}_{y,R} + \lambda^{+}_{Z,z}\chi^{(2)}_{zyx}\lambda^{+}_{y,R}\lambda^{+}_{x,R} = -i\chi^{(2)} \tag{S11}$$

$$\chi^{(2)}_{ZLL} = \lambda^{+}_{Z,z}\chi^{(2)}_{zxy}\lambda^{+}_{x,L}\lambda^{+}_{y,L} + \lambda^{+}_{Z,z}\chi^{(2)}_{zyx}\lambda^{+}_{y,L}\lambda^{+}_{x,L} = i\chi^{(2)} \tag{S12}$$

where χ^2 =100 pm/V. In a matrix form we can write:

$$\bar{\bar{\chi}}^{(2)}_{CP} = \chi^{(2)} \begin{pmatrix} \{0,0,0\} & \{0,0,i\} & \{0,i,0\} \\ \{0,0,-i\} & \{0,0,0\} & \{-i,0,0\} \\ \{-i,0,0\} & \{0,i,0\} & \{0,0,0\} \end{pmatrix} \tag{S13}$$

Thus, the nonlinear polarization source for second-harmonic light, expressed in the CP basis along the positive z-axis direction, reads as follows:

$$P^{2\omega}_{R} = 2i\varepsilon_0\chi^{(2)}E^{\omega}_{L}E^{\omega}_{Z} \tag{S14}$$

$$P^{2\omega}_{L} = -2i\varepsilon_0\chi^{(2)}E^{\omega}_{R}E^{\omega}_{z} \tag{S15}$$

$$P^{2\omega}_{z} = i\varepsilon_0\chi^{(2)}\left[(E^{\omega}_{L})^2 - (E^{\omega}_{R})^2\right] \tag{S16}$$

which are the expressions reported in Eq. 2-4 of the main text, evaluated for $\theta = 0$.

We now consider the case in which the crystalline axes are rotated with respect to the laboratory axes. In particular, we are interested in a rotation about the z-axis by an angle θ. The rotation operator that connects the crystalline frame to the rotated frame is given

by:

$$\bar{\bar{R}}_z(\theta) = \begin{pmatrix} \cos\theta & \sin\theta & 0 \\ -\sin\theta & \cos\theta & 0 \\ 0 & 0 & 1 \end{pmatrix} \tag{S17}$$

which is expressed as the following diagonal matrix in the CP basis:

$$\bar{\bar{R}}_{z,CP}(\theta) = \bar{\bar{\Lambda}}^{+}_{k,\alpha} \cdot \bar{\bar{R}}_z(\theta) \cdot \bar{\bar{\Lambda}}^{+}_{\alpha,k} = \begin{pmatrix} e^{-i\theta} & 0 & 0 \\ 0 & e^{i\theta} & 0 \\ 0 & 0 & 1 \end{pmatrix} \tag{S18}$$

Using the same procedure outlined in eq. S7 applied to the rotation matrix $R_z(\theta)$, the nonlinear susceptibility can be retrieved in the rotated frame, which, in Cartesian coordinates reads as:

$$\bar{\bar{\chi}}^{(2)}(\theta) = \chi^{(2)} \begin{pmatrix} \{0,0,\sin 2\theta\} & \{0,0,\cos 2\theta\} & \{\sin 2\theta, \cos 2\theta, 0\} \\ \{0,0,\cos 2\theta\} & \{0,0,-\sin 2\theta\} & \{\cos 2\theta, -\sin 2\theta, 0\} \\ \{\sin 2\theta, \cos 2\theta, 0\} & \{\cos 2\theta, -\sin 2\theta, 0\} & \{0,0,0\} \end{pmatrix} \tag{S19}$$

and, in the CP basis, reads as:

$$\bar{\bar{\chi}}^{(2)}_{CP}(\theta) = \chi^{(2)} \begin{pmatrix} \{0,0,0\} & \left\{0,0,ie^{-2i\theta}\right\} & \left\{0,ie^{-2i\theta},0\right\} \\ \left\{0,0,-ie^{2i\theta}\right\} & \{0,0,0\} & \left\{-ie^{2i\theta},0,0\right\} \\ \left\{-ie^{2i\theta},0,0\right\} & \left\{0,ie^{-2i\theta},0\right\} & \{0,0,0\} \end{pmatrix} \tag{S20}$$

For [110] AlGaAs, the laboratory frame and the crystalline axes are connected by the transformation matrix $\bar{\bar{T}} = \bar{\bar{R}}_z(\theta) \cdot \bar{\bar{R}}_x(\pi/4)$, where $\bar{\bar{R}}_x(\pi/4)$ is the matrix that provides a rotation of a $\pi/4$ angle about the x axis. The expression of the transformation matrix is

therefore:

$$\bar{\bar{T}} = \begin{pmatrix} \cos\theta & \frac{\sin\theta}{\sqrt{2}} & \frac{\sin\theta}{\sqrt{2}} \\ -\sin\theta & \frac{\cos\theta}{\sqrt{2}} & \frac{\cos\theta}{\sqrt{2}} \\ 0 & -\frac{1}{\sqrt{2}} & \frac{1}{\sqrt{2}} \end{pmatrix} \tag{S21}$$

in Cartesian basis and

$$\bar{\bar{T}}_{CP} = \begin{pmatrix} \frac{1}{4}\left(\sqrt{2}+2\right)e^{-i\theta} & -\frac{1}{4}\left(\sqrt{2}-2\right)e^{-i\theta} & \frac{i}{2}e^{-i\theta} \\ -\frac{1}{4}\left(\sqrt{2}-2\right)e^{i\theta} & \frac{1}{4}\left(\sqrt{2}+2\right)e^{i\theta} & -\frac{i}{2}e^{i\theta} \\ \frac{i}{2} & -\frac{i}{2} & \frac{1}{\sqrt{2}} \end{pmatrix} \tag{S22}$$

in CP basis. Performing the rotation of the tensor $\bar{\bar{\chi}}^{(2)}_{CP}(\theta)$ as in eq. S7, with a rotation defined by the operator T_{CP}, yields the nonlinear tensor of [110] AlGaAs in CP basis:

$$\bar{\bar{\chi}}^{(2)}_{[110],CP}(\theta) = \chi^{(2)} \begin{pmatrix} \left\{\frac{e^{i\theta}}{2\sqrt{2}}, \frac{e^{-i\theta}}{2\sqrt{2}}, 0\right\} & \left\{\frac{e^{-i\theta}}{2\sqrt{2}}, -\frac{3e^{-3i\theta}}{2\sqrt{2}}, 0\right\} & \left\{0, 0, -\frac{e^{-i\theta}}{\sqrt{2}}\right\} \\ \left\{-\frac{3e^{3i\theta}}{2\sqrt{2}}, \frac{e^{i\theta}}{2\sqrt{2}}, 0\right\} & \left\{\frac{e^{i\theta}}{2\sqrt{2}}, \frac{e^{-i\theta}}{2\sqrt{2}}, 0\right\} & \left\{0, 0, -\frac{e^{i\theta}}{\sqrt{2}}\right\} \\ \left\{0, 0, -\frac{e^{i\theta}}{\sqrt{2}}\right\} & \left\{0, 0, -\frac{e^{-i\theta}}{\sqrt{2}}\right\} & \left\{-\frac{e^{i\theta}}{\sqrt{2}}, -\frac{e^{-i\theta}}{\sqrt{2}}, 0\right\} \end{pmatrix} \tag{S23}$$

We can now write the nonlinear polarization in CP basis for [110] AlGaAs as:

$$P_R^{2\omega} = \frac{\varepsilon_0\chi^{(2)}}{2\sqrt{2}}\left[-3e^{-3i\theta}\left(E_L^\omega\right)^2 + 2e^{-i\theta}\left(E_R^\omega E_L^\omega - \left(E_Z^\omega\right)^2\right) + e^{i\theta}\left(E_R^\omega\right)^2\right] \tag{S24}$$

$$P_L^{2\omega} = \frac{\varepsilon_0\chi^{(2)}}{2\sqrt{2}}\left[-3e^{3i\theta}\left(E_R^\omega\right)^2 + 2e^{i\theta}\left(E_R^\omega E_L^\omega - \left(E_Z^\omega\right)^2\right) + e^{-i\theta}\left(E_L^\omega\right)^2\right] \tag{S25}$$

$$P_Z^{2\omega} = -\varepsilon_0\chi^{(2)}\sqrt{2}\left(e^{-i\theta}E_L^\omega E_Z^\omega + e^{i\theta}E_R^\omega E_Z^\omega\right) \tag{S26}$$

which provides the expressions in eq. 7-9 in the main text.

S2 Numerical simulations

Fully vectorial numerical calculations are performed in the frequency-domain with the finite element method implemented in COMSOL. The thin-layer or metasurface plane is chosen as the xy-plane, while propagation of the incident pump beam is assumed in the positive z-axis direction. For the top and bottom xy-planes, open boundaries conditions are implemented with perfectly matched layers, while periodic boundaries with Floquet condition are used for yz and xz-planes. The dispersion of AlGaAs is introduced in the numerical calculations using experimental data.[1] The second harmonic signal is calculated using a two-step procedure. First the electric field at the pump frequency is numerically calculated. Second, the optical response of the structure at the second-harmonic frequency is calculated by setting a nonlinear polarization generated by the electric field distribution at the pump frequency.

S3 Nano-chair metasurface geometry

The unit-cell of the metasurface is constituted by an AlGaAs nano-chair with an elliptical basis. The structure of the unit cell is depicted in Fig. S2. The height of the structure is 400 nm, the height of the cut is 200 nm, and the major and minor semi-axes are 325 nm and 100 nm, respectively. The metasurface periodicity is 880 nm.

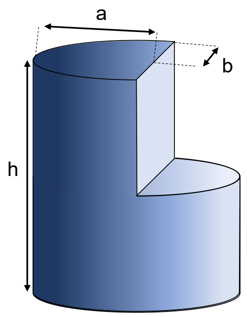

Figure S1: Drawing of the nano-chair geometry. Major and minor semi-axes are a and b, respectively. The structure height is h.

S4 Phase response of arrays with rotating crystal or meta-atom

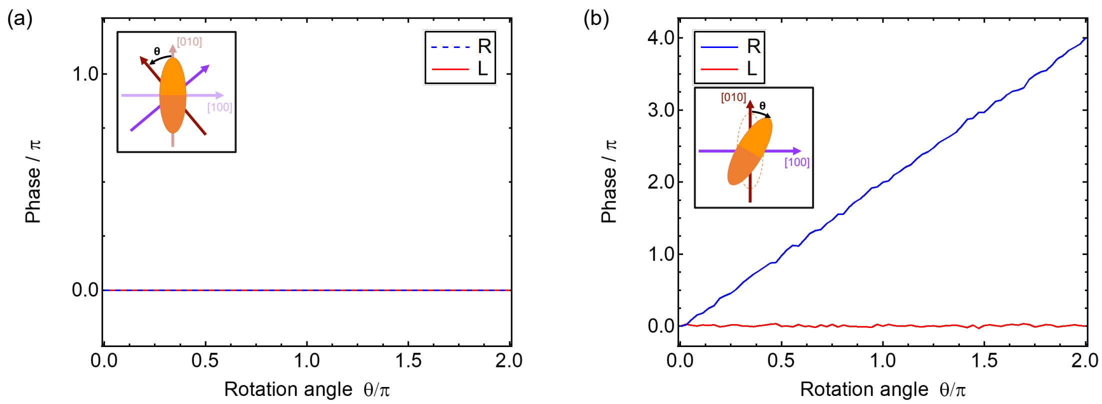

Figure S2: Phase of the transmitted light for an L illumination as a function of rotation angle θ for the case of (a) crystal rotation and (b) meta-atom rotation.

References

(1) Aspnes, D. E.; Kelso, S. M.; Logan, R. a.; Bhat, R. Optical properties of AlxGa1-x As. *Journal of Applied Physics* **1986**, *60*, 754–767.
